# Metastatic invasive lobular carcinoma of the breast to the endometrium presenting with abnormal uterine bleeding; Case report

**DOI:** 10.1016/j.amsu.2020.01.008

**Published:** 2020-02-03

**Authors:** Sardar Hassan Arif, Ayad Ahmad Mohammed, Farashin Rashid Mohammed

**Affiliations:** a General Surgeon, Department of Surgery, College of Medicine University of Duhok, DUHOK, Kurdistan Region, Iraq; b Department of Histopathology, College of Medicine University of Duhok, DUHOK, Kurdistan Region, Iraq

**Keywords:** Breast cancer, Invasive lobular carcinoma, Tamoxifen, Abnormal uterine bleeding, Laparoscopic hysterectomy

## Abstract

Metastatic cancer to the female genital organs is a very rare clinical presentation. The myometrium is more frequently affected than the endometrium. Prolonged use of tamoxifen has been found to be associated with endometrial hyperplasia, endometrial polyp formation, and the development of endometrial adenocarcinoma in some cases.

A 55-year-old lady with a history of invasive lobular carcinoma of the breast that had been operated upon with left mastectomy 7 years previously & who had also been treated with tamoxifen for 5 years presented with irregular vaginal bleeding and lower abdominal pain. The patient had a three cm uterine fibroid with an endometrial polyp. She had anemia with hemoglobin level 8 mg/dl.

Laparoscopic hysterectomy with bilateral oophorectomy was performed for a polyp in the endometrium which proved to be a metastasis from her lobular carcinoma of the breast.

Patients with breast cancer who present with abnormal vaginal bleeding should alert the physicians about the possibility of metastatic breast cancer to the uterus regardless the use of the hormonal therapy such as tamoxifen. The pathologists also should be aware of this possibility and they should examine the polyps very carefully to detect any metastatic foci especially if the patient has been treated with tamoxifen.

## Highlights

• Breast cancer is the most common type of cancer that affect women during their lifetime.
• Metastatic cancer to the female genital organs is a very rare clinical presentation.
• Abnormal vaginal bleeding in a breast cancer patient should alert to the possibility of metastatic to the uterus.

## 1 Introduction

Metastatic cancer to the female genital organs is a very rare clinical presentation and it metastasize to the ovary in most of the reported cases, metastatic cancer to the uterus is an extremely rare clinical finding and few cases are reported worldwide [1].

The myometrium is more frequently affected than the endometrium. Metastatic cancers to leiomyomas are reported in some cases [2].

Tamoxifen as an adjuvant therapy that is widely used as an adjuvant hormonal therapy in patients with hormone positive breast cancer patients. Prolonged use of tamoxifen has been found to be associated with endometrial hyperplasia, endometrial polyps formation, and the development of endometrial adenocarcinoma in some cases [3].

Patients usually present with abnormal vaginal bleeding when the tumor is affecting the endometrium or the cervix, some patients may suffer from lower abdominal pain, or the condition may be discovered during the routine gynecological examination. Combining these finding together with the previous history of breast cancer and the treatment with tamoxifen are very suggestive and should raise the suspicion about this possibility [1,4].

Patients may have elevated CA 15-3, and the transvaginal ultrasound will show the lesion, hysteroscopy or endometrial biopsy are other useful investigations that help in reaching the diagnosis [4].

The treatment is usually by hysterectomy with bilateral oophorectomy, one the diagnosis of metastatic breast cancer to the uterus have been made, search for other sites of metastatic foci must be made including local recurrence.

The work in this case report has been reported in line with the SCARE 2018 criteria [5].

## 2 Patient information

### 2.1 Clinical findings

A 55-year-old lady with a history of invasive lobular carcinoma of the breast that had been operated upon with left mastectomy 7 years previously & who had also been treated with tamoxifen for 5 years presented with irregular vaginal bleeding and lower abdominal pain.

The patient had negative drug history. The family history was negative for chronic illnesses, and psychosocial history was irrelevant.

### 2.2 Diagnostic assessment

The hemoglobin level was 8 mg/dl. Ultrasound of the uterus showed increased thickness of the endometrium, 12 mm. Hysteroscopy showed an endometrial polyp, curettage was done and the result was endometrial hyperplasia with malignant cells.

### 2.3 Therapeutic intervention

**Fig. 1:**
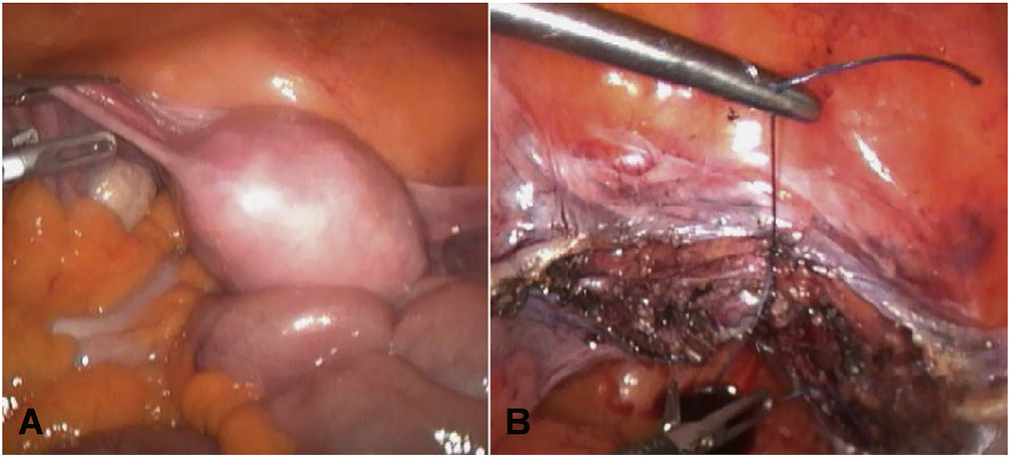
A laparoscopic view showing (A) the pelvic cavity and the pelvic organs, (B) closure of the vaginal vault after hysterectomy.

Laparoscopic hysterectomy with bilateral oophorectomy was performed for a polyp in the endometrium which proved to be a metastasis from her lobular carcinoma of the breast (Fig. 1).

**Fig. 2:**
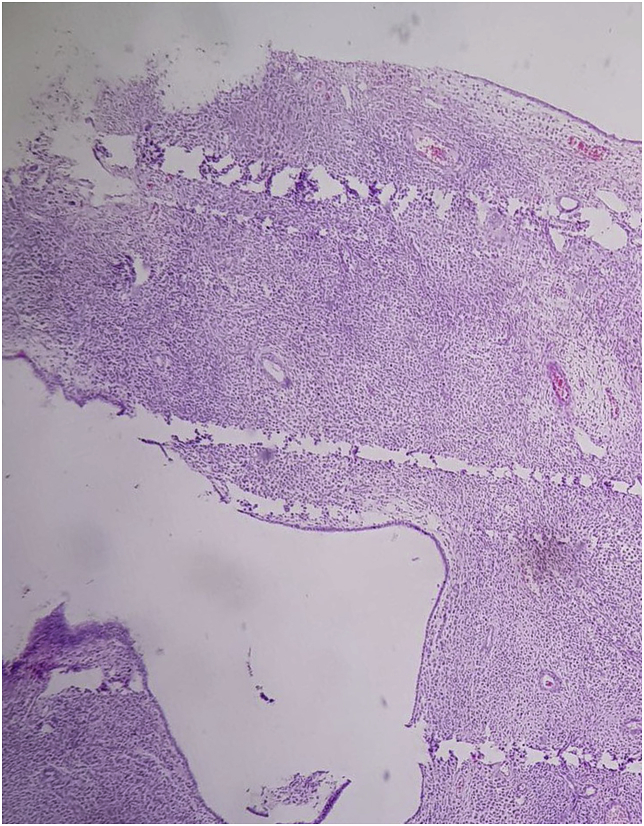
A high magnification slide showing the malignant signet ring like cells within the stroma of the endometrial polyp.

**Fig. 3:**
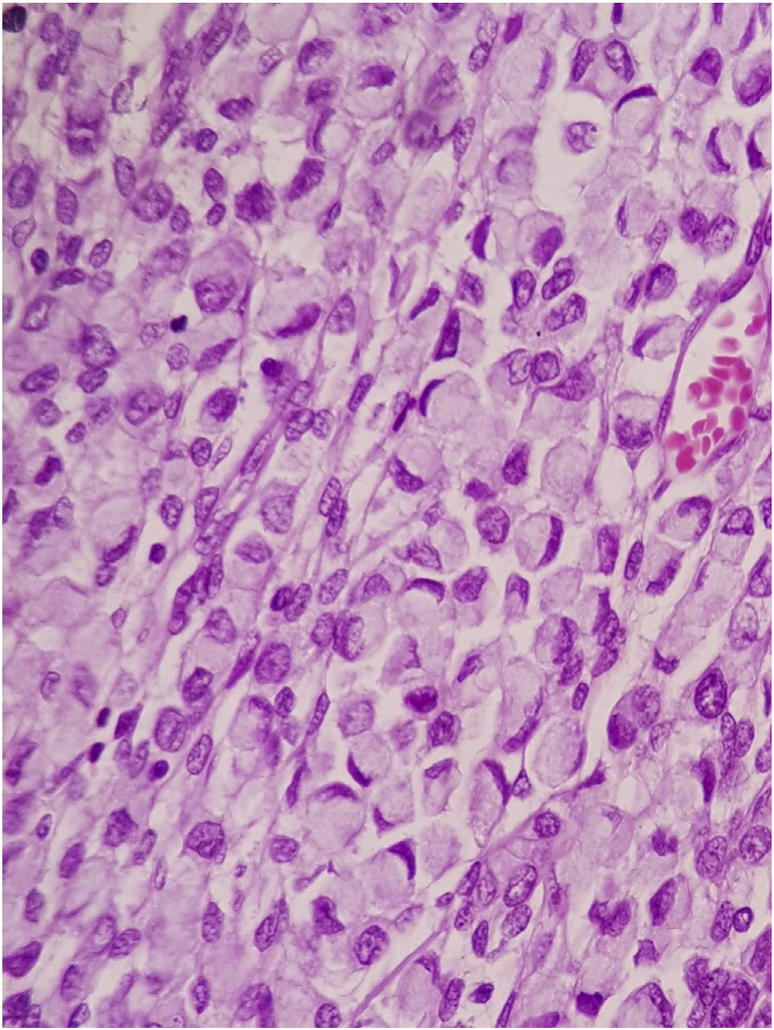
A microscopic view showing the tissue staining with CK7 (A) and GCDFP15(B).

**Fig. 4:**
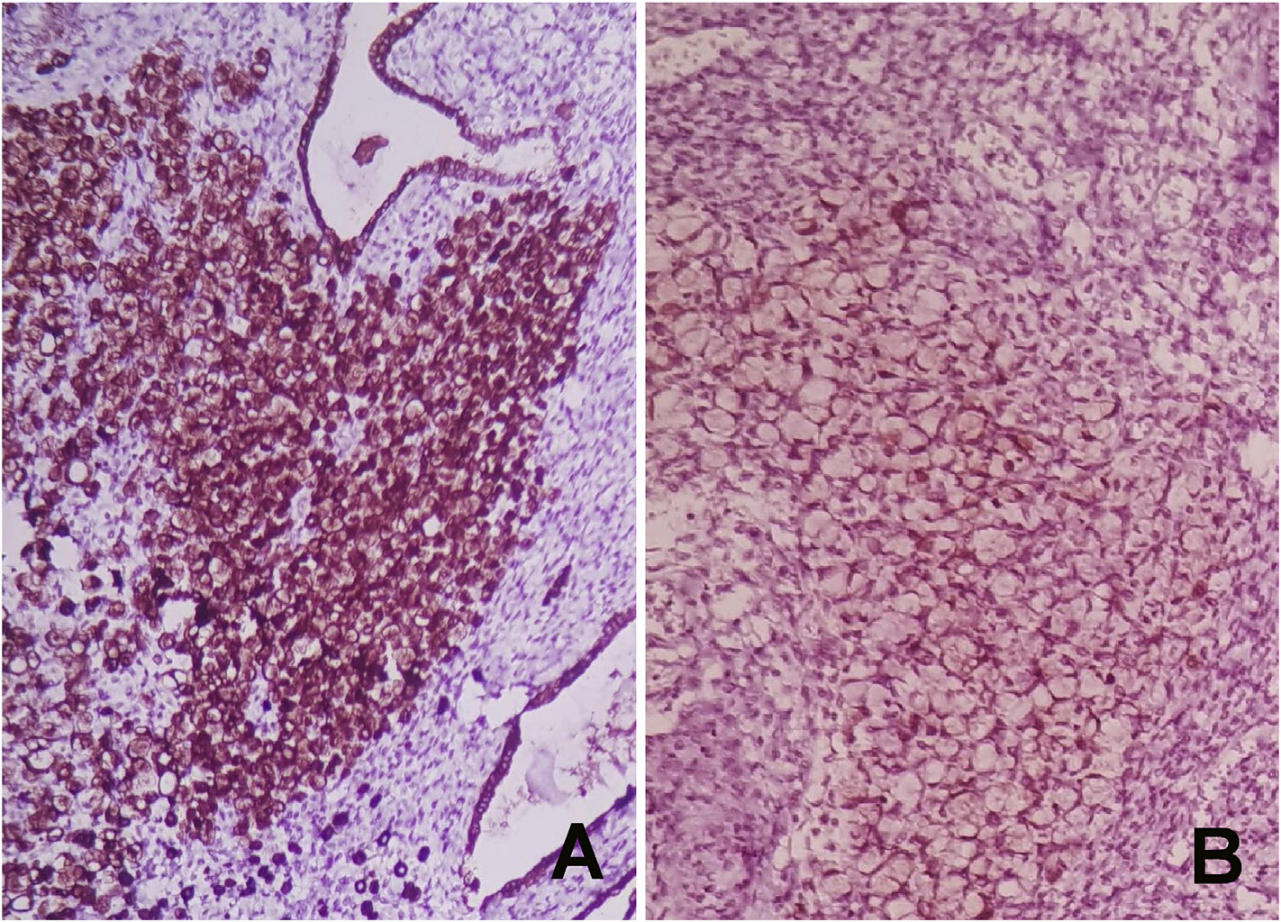
A microscopic view showing the tissue staining with CK7 (A) and GCDFP15(B).

The sample was sent for histopathological study, the report of the histopathologist showed metastatic invasive lobular carcinoma of the breast to an endometrial polyp, Fig. 2, Fig. 3, Fig. 4.

The procedure was performed by two general surgeons who are experienced in laparoscopic surgery and the tissue samples were examined by a specialist histopathologist.

### 2.4 Follow-up and outcomes

The patient was followed for 1 year after surgery with no complications and the radiological assessment showed no evidence of metastatic foci in other anatomical sites.

## 3 Discussion

Breast cancer is the commonest cancer affecting women during their life time. In a review of 63 cases who presented with metastatic cancers to the uterus, 20 lesions were discovered with biopsy samples and the rest were discovered at autopsy, metastases from breast cancer were reported in 42.9%, colon cancer in 17.5%, stomach cancer in 11.1%, pancreas in 11.1%, and the rest were from the gallbladder cancer, lung cancer, malignant melanoma, urinary bladder cancer, and thyroid cancer in order of decreasing frequency [2,6].

Some cases are reported in literature who had breast cancer treated with mastectomy with adjuvant tamoxifen therapy, they presented later with metastatic breast cancer to the uterus, most of them were invasive lobular carcinoma. The reported cases were similar to our case except that our case is presented at a younger age compared to the reported cases who were above 60 years [3].

Cases had been reported presenting with rapidly enlarging leiomyomas of the uterus due to leiomyosarcoma or the development of endometrial uterine sarcomas, the authors were unable to find a possible link whether tamoxifen was the cause or was just a coincidental finding [3].

A study was done involving 261 autopsy samples comparing the metastatic patterns between invasive ductal and invasive lobular carcinomas of the breast, there were no significant difference between the two histological types regarding the frequency of the frequency of metastatic disease, but there was significant difference in relation with the metastatic site, the commonest sites for metastasis in patients with invasive lobular carcinoma were the liver and the bones, while for invasive lobular carcinoma were the peritoneum and the retroperitoneum spaces, the hollow viscera, the genital organs, and the myocardium. This may reflect het each type of cancer has a specific predilection for metastatic potential [7].

Another retrospective analyses which involved 12 cases of metastatic breast cancer to the uterus, 10 of them were invasive lobular carcinoma, in 11 of them it was associated with ovarian metastases, and the myometrium was the most common site involved [8].

Patients with breast cancer who present with abnormal vaginal bleeding should alert the physicians about the possibility of metastatic breast cancer to the uterus regardless the use of the hormonal therapy such as tamoxifen. The pathologists also should be aware of this possibility and they should examine the polyps very carefully to detect any metastatic foci especially if the patient has been treated with tamoxifen [8].

## Ethical approval

No ethical committee approval was needed; consent have been taken from the patient to report her findings.

## Funding source

No source of funding other than the authors.

## Author contribution

The surgeon who performed the procedure: Dr Sadrar Hassan Arif and Dr Ayad Ahmad Mohammed. Study design, writing, and the final approval of the manuscript: Dr Ayad Ahmad Mohammed.

## Research registration unique identifying number (UIN)

N/A.

## Trial registry number – ISRCTN

N/A.

## Guarantor

Dr Ayad Ahmad Mohammed.

## Patient perspective

I was surprised after the surgery about the results of the histopathology, I am worried for the occurrence of new lesions in other parts of my body.

## Informed consent

An informed written consent was taken from the patient for reporting this case and the accompanying images.

## Provenance and peer review

Not commissioned, externally peer reviewed.

## Declaration of competing interest

No conflicts of interest present.
